# EphA3 targeted by miR-3666 contributes to melanoma malignancy via activating ERK1/2 and p38 MAPK pathways

**DOI:** 10.1515/med-2022-0597

**Published:** 2022-12-16

**Authors:** Di Ming, Jingjing Ma

**Affiliations:** Dermatological Department, Wuhan Asia General Hospital, Economic and Technological Development Zone, Wuhan 430056, Hubei, China; Dermatological Department, Wuhan Asia General Hospital, No. 300 Taizihu North Road, Economic and Technological Development Zone, Wuhan 430056, Hubei, China

**Keywords:** melanoma, EphA3, miR-3666, proliferation, migration

## Abstract

Melanoma is a rare, fatal type of skin tumor. Although EPH receptor A3 (EphA3) is deregulated in melanoma, its detailed role remained uncharacterized. Using real time quantitative PCR analysis and western blotting, EphA3 was identified to be upregulated in melanoma tissues and cells, while miR-3666 showed an opposite expression trend. Cell counting kit-8, scratch wound, and *in vivo* assays proved that EphA3 silence inhibited the melanoma cell proliferation and migration and retarded tumor growth *in vivo*. Furthermore, western blotting results displayed that EphA3 silence resulted in a low expression of p38-MAPK and p-ERK1/2. Mechanically, miR-3666 was proved to target EphA3 3′UTR by the luciferase reporter assay. Furthermore, miR-3666 mimic compromised the driven melanoma cell proliferation and migration by EphA3 overexpression. In addition, induction of ERK1/2 and p38 MAPK pathways offset the positive effect of EphA3 overexpression on melanoma cells. In conclusion, miR-3666 downregulated EphA3 expression and retarded melanoma malignancy via inactivating ERK1/2 and p38 MAPK pathways. Hence, miR-3666/EphA3 axis may represent a druggable target against melanoma progression.

## 1 Introduction

Melanoma is a kind of fatal cutaneous malignancy characterized by exceptional aggressiveness and metastasis. Global Cancer Statistics 2020 estimated about 324,635 new cases and 57,043 new death in 2021 [1]. Ultraviolet ray radiation, advanced age, and genetic predisposition caused the exacerbation burden of melanoma [2]. Though great strides in melanoma treatment have achieved in high-risk resectable melanoma, the overall prognosis remains inferior [3]. This consequence might be linked with the lack of prompt diagnosis and intervention due to silent and clinical presentation of melanoma. Therefore, understanding the mechanism behind melanoma progression might promote the diagnosis and treatment of melanoma.

EPH receptor A3 (EphA3) is one member of the ephrin receptor subfamily of the protein-tyrosine kinase family. EPH and EPH-related receptors have been implicated in mediating developmental events, particularly in the nervous system. Hitherto, its role in cancers gains attention from scientists due to its tumor-promoting and tumor-suppressive role in different cancers. For example, loss of EphA3 expression is detected in colorectal cancer and verified as a tight linkage with advanced pathological feature of patients [4]. Overexpressing of EphA3 represses the esophageal squamous cell carcinoma cell growth *in vitro* [5]. On the contrary, upregulation of EphA3 activates AKT signaling pathway and then facilitates glioblastoma progression [6]. The bispecific antibody against EphA3 lessens the tumorigenesis of recurrent glioblastoma by downregulating ERK signaling pathway [7]. Therefore, the role of EPHA3 in cancers presents context dependence. In melanoma, a genomic investigation about melanoma by Timar et al. suggests that EphA3 is a marker gene of melanoma [8]. However, its role and associated mechanism remained elusive.

microRNA (miRNA) belongs to the non-coding transcripts, characterizing a size of 20 nt. Compelling evidence has shown that it could reshape the cellular protein expression by interacting with its targeted mRNA and stepwise influence different cell process [9]. Activities of miRNAs are also pinpointed in different cancers, including melanoma. For instance, miR-137 targeting solute carrier family 1 member 5 regulates ferroptosis in melanoma and exerts tumor-promotion effect [10]. MiR-204-5p actively participates vemurafenib resistance in melanoma by activating ERK1/2 signaling pathway [11]. As for miR-3666, its tumor-suppressive function has been elucidated in different cancers, such as ovarian carcinoma, glioblastoma, and thyroid carcinoma [12,13,14]. And yet, its role in melanoma remains under investigation. By the bioinformatics analysis, we found the potential interaction between miR-3666 and EphA3. More importantly, miR-3666 inactivated ERK1/2 and p38 MAPK signaling pathways to suppress colorectal cancer [15]. Therefore, we speculated that miR-3666/EphA3 might have inhibitory roles in melanoma cells by regulating ERK1/2 and p38 MAPK signaling pathways.

Together with literature review and bioinformatics analysis, we hypothesize that miR-3666 is downregulated in melanoma and inhibits melanoma development by targeting EphA3. Herein, our aim is to explore how EphA3 impacts melanoma *in vitro* and *in vivo* and identify the interaction of miR-3666 and EphA3 on melanoma cells. Our findings might offer novel insight for melanoma progression.

## 2 Methods

### 2.1 Clinical samples

A total of 45 paired melanoma tissues and corresponding normal tissues were surgically obtained from melanoma patients first diagnosed in Wuhan Asia General Hospital. The clinical samples were snap frozen in liquid nitrogen and stored in −80°C for the next gene quantification.

### 2.2 Cell cultivation, transfection, and treatment

Human melanoma cells (A375, A875, and A2058) and human immortalized epidermal HaCAT cells were purchased from BeNa culture collection (BNCC, China). HaCAT cells (T25 medium, ThermoFisher, USA), A375 (RPMI-1640, ThermoFisher), and A875 and A2058 (CM1-1 medium, ThermoFisher) were cultivated with 10% fetal bovine serum (ThermoFisher) and 1% penicillin/streptomycin in the 5% CO_2_ atmosphere at 37°C.

Si-EphA3 and si-NC, pcDNA-EphA3 (EphA3-OE) and the empty vector, miR-3666 mimic and mimic NC were obtained from Gema (Shanghai, China). These vectors were introduced into A375 and A875 cells with Lipo 3000 reagent (ThermoFisher). The transfection efficiency was determined by real time quantitative PCR (RT-qPCR) after 48 h post-transfection.

ERK1/2 inhibitor (PD98059) and p38 MAPK inhibitor (SB203580) purchased from Cell Signaling Technologies (USA) were dissolved in dimethyl sulfoxide (DMSO), respectively. A375 and A875 cells were pretreated with 10 μM SB203580 or 30 μM PD98059 for 2 h before EphA3-OE stimulation. The control group was treated with equivalent DMSO [16].

Lentivirus-Sh-EphA3 and Lentivirus-sh-NC were purchased from Gema (China). After amplification in 293T cells by calcium phosphate transfection (ThermoFisher), the filtered lentiviral particles were infected to A375 cells with Lipo 3000 reagent (ThermoFisher). Forty-eight hours later, the cells carrying viral particles were subjected to puromycin (P8833; Sigma-Aldrich) selection for 14 days. The survivals were amplified and collected for RT-qPCR verification.

### 2.3 RT-qPCR

Total RNAs were extracted using a RNA isolation kit (MEFx Translational Medicine Co., Ltd, China). Construction of cDNA was implemented via SuperScript IV CellsDirect cDNA synthesis kit (ThemoFisher) or One-Step microRNA cDNA Synthesis Kit (Beijing Baiao Lai Bo Technology Co., Ltd, China). RT-qPCR analysis was performed on AB7500 fast platform (Applied Biosystems, USA) using 2× SYBR Green Mix (ThermoFisher). The data were normalized to GAPDH (for mRNA) or U6 (for miRNA) using the Delta-Delta CT method [17]. The primers are listed in Table 1.

**Table 1:** Real-time PCR primer synthesis list

Gene	Sequences	
EphA3	Forward	5′-CCCGCCTCACAGTTCTACTC-3′
	Reverse	5′-ATCTTTTGCAAGATCCCTGCC-3′
miR-3666	Forward	5′-AATGAGACCCAGTGCAAGTGT-3′
	Reverse	5′-GACAGAGACCAGGCAGTGTG-3′
U6	Forward	5′-CTCGCTTCGGCAGCACA-3′
	Reverse	5′-AACGCTTCACGAATTTGCGT-3′
GAPDH	Forward	5′-AGAAAAACCTGCCAAATATGATGAC-3′
	Reverse	5′-TGGGTGTCGCTGTTGAAGTC-3′

### 2.4 Western blot

Protein samples were obtained from A375 and A875 cells exposed to RIPA lysis buffer (Solarbio, China). BCA assay kit (Abcam, USA) was adopted to assess the protein concentration. Proteins (20 μg per lane) were resolved in 12% sodium dodecyl sulfate polyacrylamide gel electrophoresis and detected on poly(vinylidene fluoride) membranes after which was blocked by 5% skim milk powder at room temperature for 2 h. Protein detection on membranes was detected with anti-p38MAPK (FNab06077, 1:1,000, FineTest, Biological Technology Co., Ltd, China), anti-p-p38MAPK (44-684G, 1:1,000, ThermoFisher), anti-p-ERK1/2 (44-680G, 1:1,000, ThermoFisher), anti-ERK1/2 (13-6200, 1:1,000,ThermoFisher), anti-EphA3 (310438-T08, 1:1,000, SinoBiological, China), and anti-GAPDH (100242-T08, 1:1,000, SinoBiological) at 4°C for 24 h. Next, horseradish peroxidase-conjugated secondary antibodies (A32731, 1:1,000, ThermoFisher) were added and incubated with these membranes at room temperature for 1 h. Immune blots were developed with enhanced chemiluminescence (GE Healthcare, USA) and counted quantitatively by ImageJ.

### 2.5 Cell counting kit-8 (CCK-8) assay

A375 and A875 cells (2.5  ×  10^5^ cells/well) received CCK-8 reagent (10 μL, ThermoFisher) treatments for 4 h after both cells on 96-well plates underwent cultivation for 0, 24, 48, and 72 h, respectively. The optical density values of 450 nm were read using a microplate (Bio-Rad, USA).

### 2.6 Scratch wound assay

A375 and A875 cells (2.5  ×  10^4^ cells/well) were seeded on six-well culture plates. When both cells were 100% confluence, wound gaps were scratched using a P200 pipette tip. Cells were washed thrice with 1× phosphate-buffered saline and photographed. After additional 24 h culture, the wound areas were captured and analyzed using ImageJ software (NIH, USA).

### 2.7 Tumorigenic assessment *in vivo*

BALB/c nude mice (4–5 weeks old, about 20 g) were purchased from the Wuhan University Center for Animal Experiment/Animal Biosafety Level III laboratory (ABSL-III lab) of Wuhan University (Wuhan, Hubei, China). All mice were supplied with autoclaved food and water with 12-h dark and 12-h light. The mice received a subcutaneous injection of A375 cells (10^7^ cells/mouser) stably with sh-EphA3 or sh-NC in their flanks. Every 4 days, tumor size was measured and tumor volume was calculated as: (tumor volume  =  length × width^2^/2). At post-injection of 28 days, these mice were asphyxiated with CO_2_. The tumors were removed and measured. All the animal procedure in this research was conducted with the approval of Wuhan Asia General Hospital.

### 2.8 Luciferase reporter assay

The target site of miR-3666 to EphA3 3′UTR was named wild-type (WT) EphA3 3′UTR. The corresponding mutant (MUT) EphA3 3′UTR were obtained using the QuikChange Mutagenesis Kit (Stratagene, USA). The EphA3 3′UTR-WT and EphA3 3′UTR-MUT sequences were amplified and fused into pGL3 Luciferase Reporter Vectors (Promega, USA). The resulted pGL3-EphA3 3′UTR-MUT vectors or EphA3 3′UTR-WT vectors were co-transfected with miR-3666 mimic or mimic NC into A375 and A875 cells. Forty-eight hours post-transfection, the luciferase activities were quantified by luciferase reporter system (Promega).

### 2.9 Statistical analysis

GraphPad Prism 9.0 (GraphPad Prism Software, USA) was employed to analyze the outcome expressed mean ± SD. Student’s *t*-test was utilized to compare the findings from two groups. One-way analysis of variance was implemented for multiple groups comparison accompanied with Dunnet’s tests. Pearson’s analysis was used to analyze the correlation of miR-3666 and EphA3 in melanoma tissues. Results were considered significant if *P* < 0.05.


**Ethics approval:** This research has been approved by the Ethics Committee of the Wuhan Asia General Hospital (Wuhan, China). The processing of clinical samples is in strict compliance with the ethical standards of the Declaration of Helsinki. All patients provided their written informed consent. The procedures executed in the animal study were approved by Wuhan Asia General Hospital. All animal experiments comply with the ARRIVE guidelines.
**Consent to participate:** All patients signed a written informed consent.

## 3 Results

### 3.1 Upregulation of EphA3 in melanoma tissues and cells

**Figure 1:**
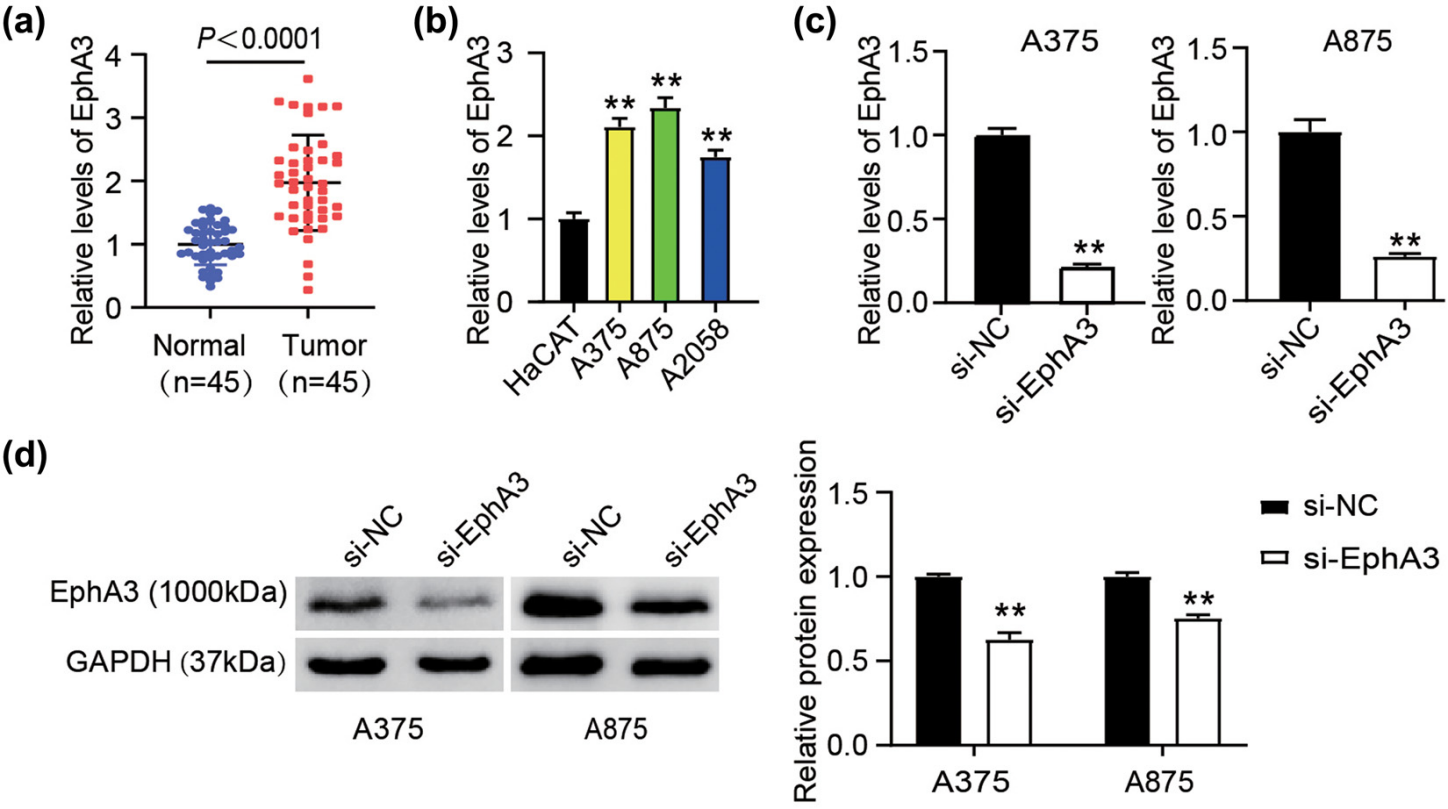
Upregulation of EphA3 in melanoma tissues and cells. (a) EphA3 mRNA levels were detected in melanoma tissues and normal tissues by RT-qPCR analysis. (b) EphA3 mRNA levels were detected in a pan of melanoma cells (A375, A875, and A2058) and HaCAT cells by RT-qPCR analysis. *P < 0.01 vs HaCAT. (c) Si-EphA3 or si-NC was transfected into A375 and A875 cells. Forty-eight hours post-transfection, phA3 mRNA levels were detected in these transfected cells using RT-qPCR analysis. **P < 0.01 vs si-NC. (d) Si-EphA3 or si-NC was transfected into A375 and A875 cells. Forty-eight hours post-transfection, EphA3 proteins were detected in these transfected cells using western blot analysis. **P < 0.01 vs si-NC.

To confirm the previous findings that the EphA3 is associated with melanoma malignancy [18], we measured its expression in melanoma tissues and cells. Undeniably, highly expressed EphA3 was measured in melanoma tissues (Figure 1a). Similarly, in the three melanoma cells (A375, A875, and A2058), the high expression of EphA3 was confirmed compared to HaCAT cells (Figure 1b). Especially the A375 and A875 cells exhibited an approximate two-fold increment of EphA3 mRNA levels. Consequently, we chose both melanoma cells for following cell functional verification. Subsequently, we successfully knocked down EphA3 expression in A375 and A875 cells with sh-EphA3 (Figure 1c and d). Therefore, EphA3 expression was abundant in melanoma.

### 3.2 EphA3 silencing curbs the melanoma cell proliferation and migration *in vitro* by the impairment of MAPK/ERK signaling pathway

**Figure 2:**
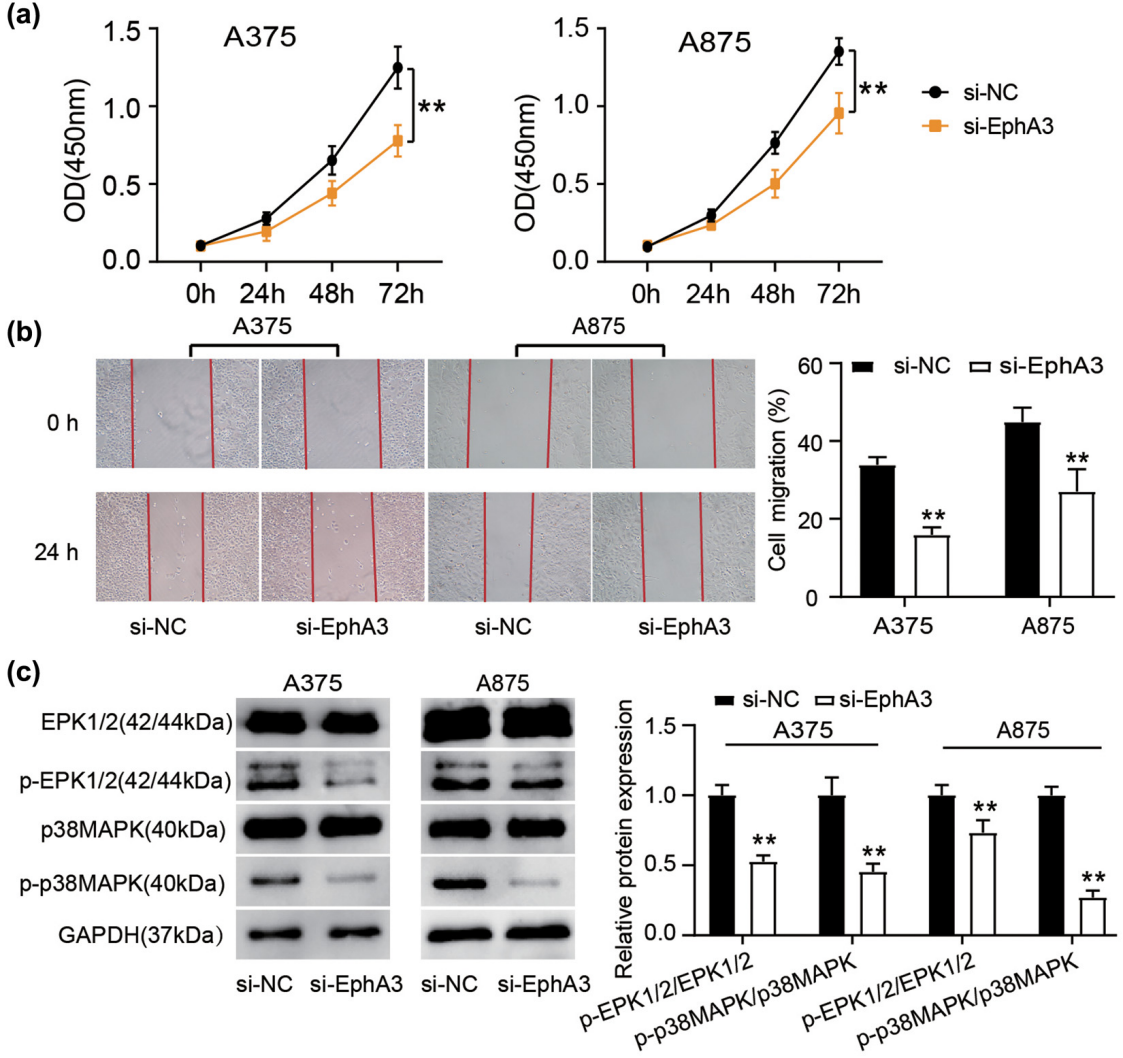
EphA3 silencing curbs the melanoma cell proliferation and migration *in vitro* by the impairment of MAPK/ERK signaling pathway. Si-EphA3 or si-NC was transfected into A375 and A875 cells. Forty-eight hours post-transfection. (a) Cell proliferation was assessed by CCK-8 assays. (b) Scratch wound assays were adopted to determine the melanoma cell migration. (c) Western blot analysis of ERK1/2, p-ERK1/2, p38MAPK, and p-p38MAPK in these transfected cells. **P < 0.01 vs si-NC.

Based on above results, upon EphA3 silence, evident impairments in cell proliferation were detected in both melanoma cells (Figure 2a). A parallel decrement in cellular migration was also manifested in A375 and A875 cells transfected with si-EphA3 compared to si-NC (Figure 2b). To mine the behind mechanism of EphA3-driven melanoma progression, we tested whether EphA3 silence impacts the ERK signaling cascade since anti-EphA3 antibodies could retard glioblastoma progression by modifying ERK1/2 signaling pathway [7]. Unsurprisingly, a compelling decrement of p-ERK1/2 and p-p38 MAPK protein levels was detected in A375 and A875 cells when EphA3 silence (Figure 2c). Therefore, the activation of ERK1/2 and p38 MAPK signaling pathways plays an important role in EphA3-driven melanoma cell proliferation and migration.

### 3.3 EphA3 silencing attenuates the tumorigenic potential in melanoma *in vivo*

**Figure 3:**
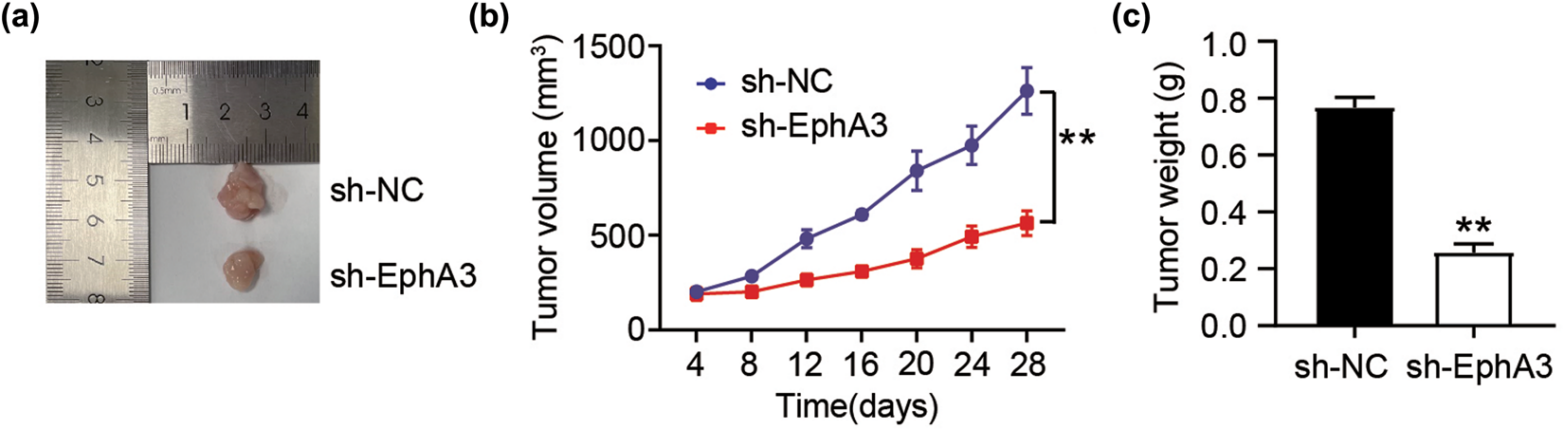
EphA3 silencing attenuates the tumorigenic potential in melanoma *in vivo*. A375 cells (10^7^ cells/mouser) carrying sh-EphA3 or sh-NC were subjected to subcutaneous flank injection to mouse (*n* = 5). (a) Representative pictures of tumor growth. (b) Tumor weight was tested every 4 days. (c) At 28 h post-injection, the tumors were resected and weight. **P < 0.01 vs sh-NC.

Having verified the function of EphA3 *in vitro*, we next examined if EphA3 is necessary for melanoma malignancy *in vivo*. To address this question, we subcutaneously injected A375 cells stably transfected with sh-EphA3 or sh-NC into nude mice. As shown in Figure 3a–c, the tumor weight and size were strongly arrested upon EphA3 silence. Therefore, EphA3 silence decelerated the cancerogenic potential of melanoma *in vivo*.

### 3.4 EphA3 is a target gene of miR-3666

**Figure 4:**
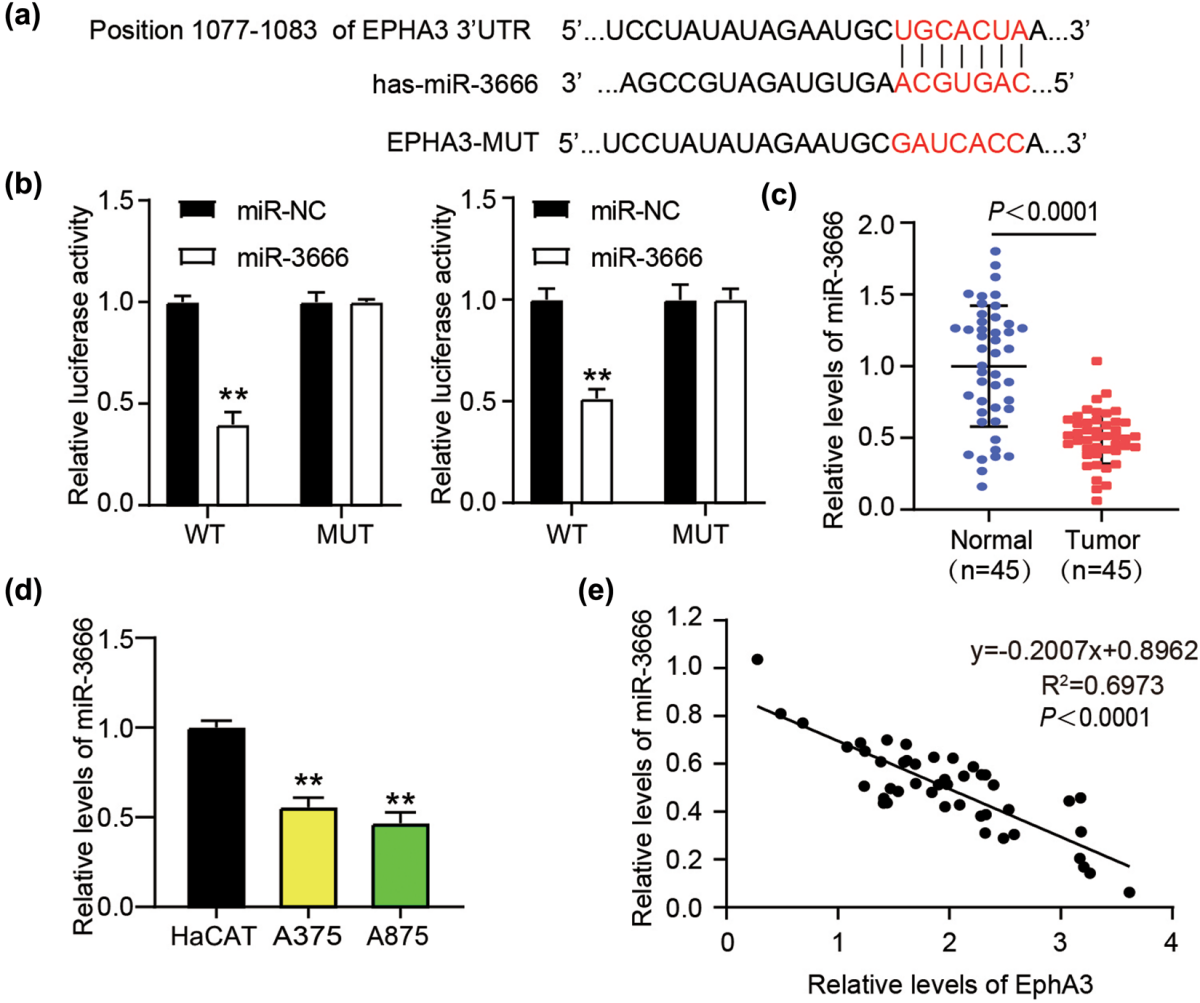
EphA3 is targeted by miR-3666. (a) The base pairing between EphA3 3′UTR and miR-3666 was predicted by starBase analysis. (b) Luciferase activity of EphA3 3′UTR-WT or EphA3 3′UTR-MUT driven was assessed by luciferase system in A375 and A875 cells’ co-transfection with miR-3666 mimic or mimic NC. **P < 0.01 vs miR-NC. (c) RT-qPCR analysis of miR-3666 expression in melanoma tissues and normal tissues. (d) RT-qPCR analysis of miR-3666 expression in melanoma cells (A375 and A875) and HaCAT cells. **P < 0.01 vs HaCAT. (e) Pearson analysis of the correlation between EphA3 and miR-3666.

Considering miRNA-mediated gene silencing, we next interrogated the upstream miRNAs controlling EphA3 expression and stepwise control cellular processes. Target prediction by starBase analysis showed that miR-3666 recognized the 3′UTR sequence of EphA3 (Figure 4a). To further clarify the base pairing between miR-3666 and EphA3 3′UTR, we constructed EphA3 3′UTR-WT and EphA3 3′UTR-MUT luciferase reporter vectors and then co-introduced them into A375 and A875 cells with miR-3666 mimic or mimic NC. This target recognition was further evidenced by that miR-3666 mimic diminished the EphA3 3′UTR-WT-derived luciferase activity while showed no impact on the EphA3 3′UTR-WT derived (Figure 4b). Furthermore, the low expression of miR-3666 was tested in melanoma tissues (Figure 4c) and cells (Figure 4d). In addition, the negative correlation between miR-3666 expression and EphA3 in clinical samples (Figure 4e) was further corroborated this miRNA-mRNA interaction. Taken together, miR-3666 directly targeted EphA3 and negatively regulated its expression.

### 3.5 MiR-3666 overexpression abrogates the EphA3-driven proliferation and migration in melanoma cells through ERK1/2 and p38 MAPK signaling pathways

**Figure 5:**
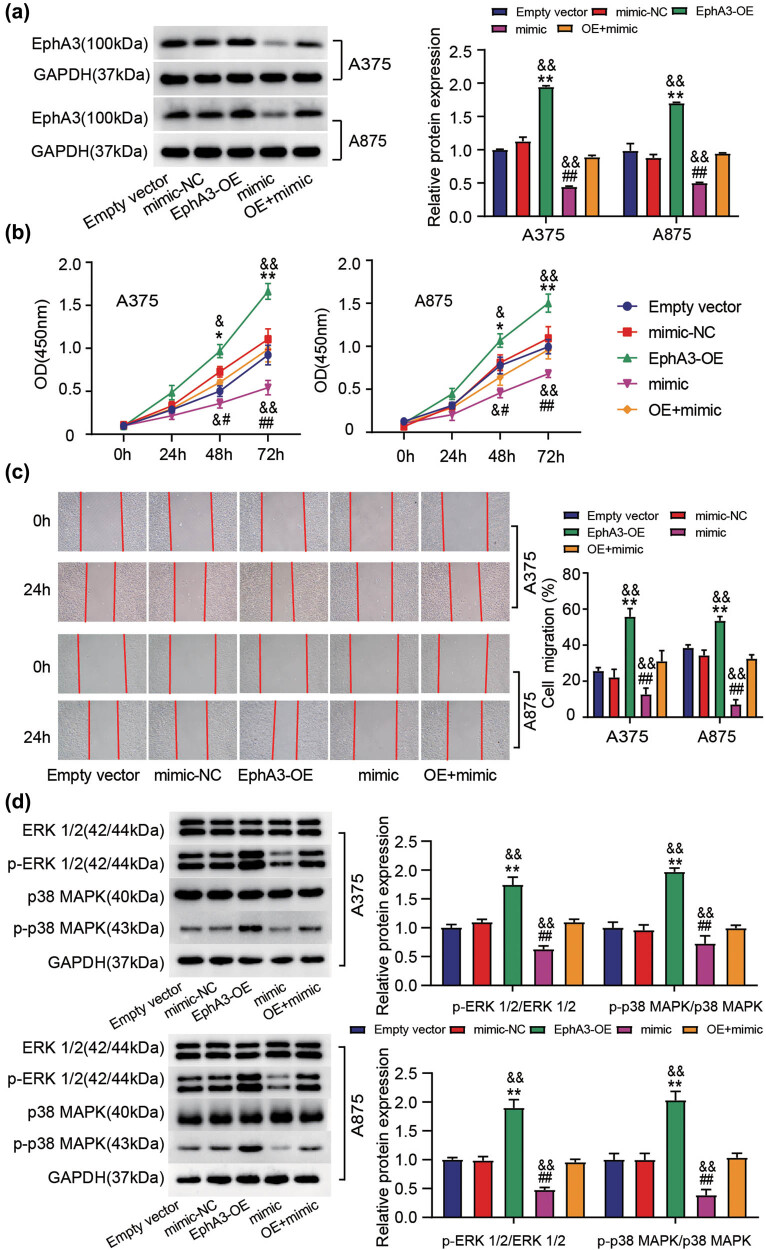
MiR-3666 overexpression abrogates the EphA3-driven proliferation and migration in melanoma cells through MAPK/ERK signaling pathway. Empty vectors, EphA3 overexpressing vectors (EphA3-OE), miR-3666 mimic (mimic), mimic NC, and EphA3-OE+ mimic were introduced into A375 and A875 cells. After 48 h transfection, (a) Western blot analysis of EphA3 expression in these above transfected cells. (b) Proliferation in these above transfected cells was determined by CCK-8 assay. (c) Migration in these above transfected cells was assessed by scratch wound assays. (d) Western blot analysis of ERK1/2, p-ERK1/2, p38MAPK, and p-p38MAPK expressions in these above transfected cells. *P < 0.05, **P < 0.01 vs empty vector; ^#^ *P* < 0.05, ^##^ *P* < 0.01 vs mimic NC; ^&^ *P* < 0.05, ^&&^ *P* < 0.01 vs OE + mimic. OE + mimic: EphA3-OE + mimic.

To verify the functional relevance of miR-3666 with EphA3, we delivered EphA3-OE vectors and miR-3666 mimic into A375 and A875 cells. As shown in Figure 5a, transfection of miR-3666 mimic diminished the EphA3 expression resulted from the transfection of EphA3-OE vectors. Functionally, EphA3 overexpression strengthened the proliferation and migration of A375 and A875 cells; on the contrary, miR-3666 mimic weakened two malignant behaviors of melanoma cells; more importantly, the promotion of EphA3 overexpression on melanoma cell growth was blockaded by miR-3666 mimic (Figure 5b and c). Western blot analysis further showed that the EphA3-induced upregulation of p-ERK1/2 and p-p38 MAPK was abrogated by miR-3666 mimic in melanoma cells (Figure 5d). Therefore, miR-3666 could sponge EphA3 to exert its inhibitory effect on melanoma malignancy.

### 3.6 The inactivation of ERK1/2 and p38 MAPK signaling pathways reverses the EphA3-driven proliferation and migration in melanoma cells

**Figure 6:**
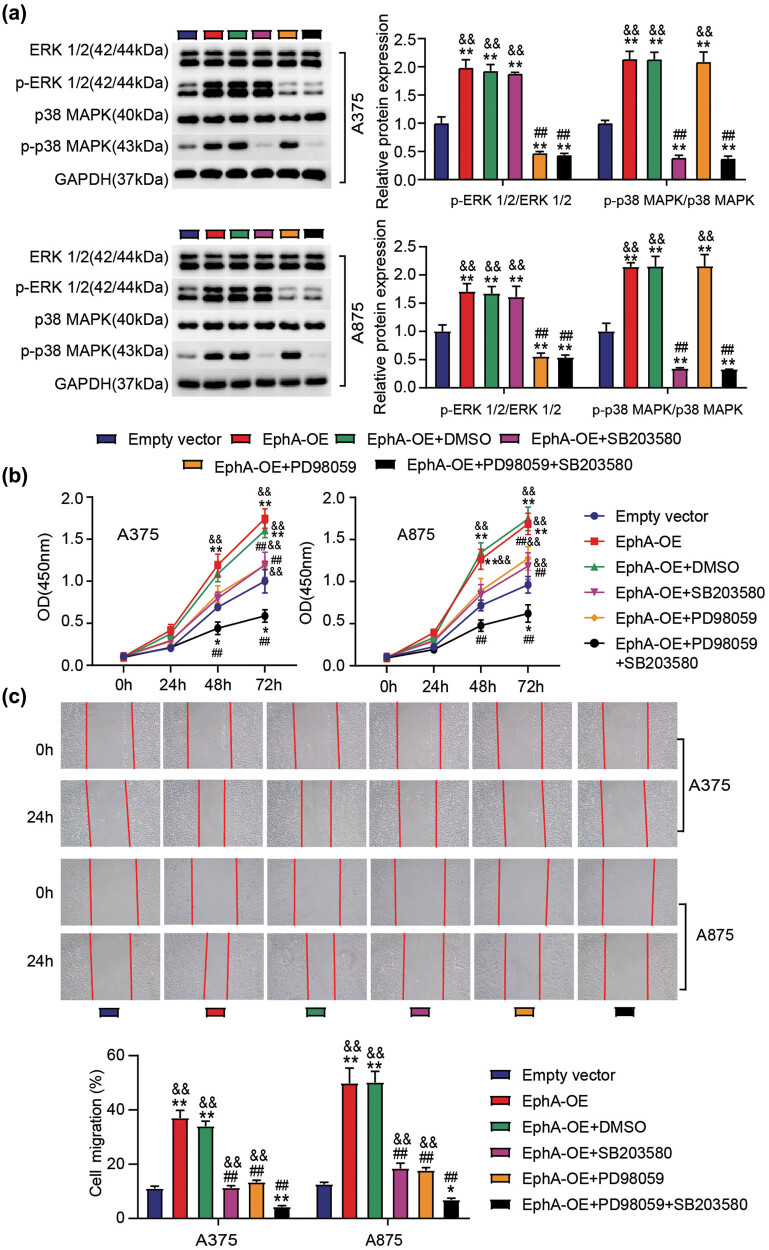
The inactivation of ERK1/2 and p38 MAPK signaling pathways’ reverses the EphA3-driven proliferation and migration in melanoma cells. A375 and A875 cells transfected with empty vectors or EphA3 overexpressing vectors (EphA3-OE) were pretreated with D98059 (ERK1/2 inhibitor) or SB203580 (p38 MAPK inhibitor). After 48 h transfection, (a) Western blot analysis of ERK1/2, p-ERK1/2, p38MAPK, and p-p38MAPK expressions in these above transfected cells. (b) Proliferation in these above treated cells was determined by CCK-8 assay. (c) Migration in these above treated cells was assessed by scratch wound assays. *P* < 0.05, **P < 0.01 vs empty vector; ^##^ *P* < 0.01 vs EphA-OE; ^&&^ *P* < 0.01 vs EphA-OE + PD98059 + SB203580.

Next, we verified the functional correlation between EphA3 and ERK1/2 or P38 MAPK signaling pathways. As shown in Figure 6a, PD98059 (ERK1/2 inhibitor) and SB203580 (p38 MAPK inhibitor) treatment inhibited p-ERK1/2 and p-p38 MAPK protein levels induced by EphA3 overexpression, respectively. In addition, PD98059 and SB203580 could partially attenuate the enhanced effects of EphA3 overexpression on the proliferation and migration of A375 and A875 cells, respectively, and PD98059 combined with SB203580 could further inhibit the effect of EphA3 overexpression (Figure 6b and c). Therefore, the carcinogenesis of EphA3 on melanoma may be mediated through the activation of ERK1/2 and P38 MAPK signaling pathways.

## 4 Discussion

In our present work, we found that EphA3 silence inhibited melanoma cells’ proliferation and migration by inactivating ERK1/2 and p38 MAPK signaling pathways. The tumor suppression of EphA3 silence in melanoma *in vitro* was further validated by *in vivo*. Mechanically, miR-3666 could abrogate the pro-proliferation and pro-migration effect of EphA3 overexpression on melanoma cell growth by inactivating ERK1/2 and p38 MAPK signaling pathways. Collectively, EphA3 targeted by miR-3666 contributes to melanoma malignancy via activating ERK1/2 and p38 MAPK pathways.

Previous investigations have showed that EphA3 has different roles in different cancers [19]. For example, EphA3 upregulation stimulates angiogenesis in multiple myeloma and attenuates cancer progression [20]. And yet, the downregulation of EphA3 protein is detected in clear-cell renal cell carcinoma and negatively associated with tumor diameter and advanced stage of patients [21]. In melanoma progression, somatic and germline mutations of EphA3 have been detected [22]. Furthermore, irradiation treatment could lessen malignant behaviors of human melanoma cells in parallel with the downregulation of EphA3 [23]. However, its detailed role in melanoma is still unknown. Our data showed that EphA3 was highly expressed in melanoma tissues and cells, and EphA3 silence mitigated the tumorigenic potential of melanoma *in vitro* and *in vivo*. And EphA3 overexpression boosted melanoma cell proliferation and migration *in vitro*. In glioma stem cells, EphA3 is responsible for sustained ERK1/2 activation and promotes its differentiation [24]. Hence, we test the ERK1/2 and p38 MAPK signaling pathways in melanoma cell when EphA3 silence. The outcome showed that EphA3 silence disrupted sustained ERK1/2 and p38 MAPK activation. In a word, EphA3 promoted the malignant phenotypes of melanoma through driving ERK1/2 and p38 MAPK signaling pathways.

miRNA regulation plays an important role in shaping the cellular protein landscape by targeting mRNA 3′UTR [25]. Subsequent prediction showed that miR-3666 target EphA3 3′UTR, which is validated by luciferase reporter assays. Previously, miR-3666 performs its tumor-suppressive action in different cancers. For example, miR‑3666 suppressed the uncontrolling proliferation and migration of colorectal cancer cells [26]. The well-known oncoprotein of STAT3 (signal transducer and activator of transcription 3) is downregulated by miR‑3666, resulting in the retention of ovarian carcinoma progression [12]. Consistently, for the first time, we also found tumor suppression in miR‑3666 overexpressing melanoma cells, evidenced by the reduction of cell proliferation and migration along with the transfection of miR-3666 transfection. Notably, miR-3666 is reportedly responsible for the disruption of ERK1/2 and p38 MAPK signaling pathways in colorectal cancer [15]. Congruently, miR-3666 transfection blunted the ERK1/2 and p38 MAPK signaling pathways. Therefore, miR-3666 might inactivate ERK1/2 and p38 MAPK signaling pathways to retard the melanoma progression. As its interaction with EphA3, miR-3666 mimic abrogated the increased proliferation and migration of melanoma cells driven by EphA3 overexpression through ERK1/2 and p38 MAPK signaling pathways. Therefore, miR-3666 downregulates EphA3 expression and suppresses melanoma malignancy via suppressing ERK1/2 and p38 MAPK pathways.

## 5 Conclusion

In summary, our results showed that miR-3666 targeting EphA3 downregulated EphA3 expression and thereby suppressed the melanoma cell proliferation and migration through disruption of ERK1/2 and p38 MAPK signaling pathways. Our findings suggest that miR-3666/EphA3 axis might be an effective target for melanoma intervention. However, this study is limited as the small sample size. Furthermore, EphA3’s function might be regulated by different miRNAs because of different base pairs. Therefore, more detailed mechanism of EphA3 driven should be explored in the future.
